# *Campylotropis anningensis* (Fabaceae, Papilionoideae), a new species from Yunnan, China

**DOI:** 10.3897/phytokeys.277.199754

**Published:** 2026-07-22

**Authors:** Li-Sha Jiang, Bo Pan, Jin-Yi Zhu, Xiong Li, Kai-Wen Jiang, Li-Chun Yan, Cheng-Wu Liu, En-De Liu, Bo Xu

**Affiliations:** 1 China-Croatia Belt and Road Joint Laboratory on Biodiversity and Ecosystem Services, Mountain Ecological Restoration and Biodiversity Conservation Key Laboratory of Sichuan Province, Key Laboratory of National Forestry and Grassland Administration on Biodiversity Conservation on the Qinghai-Xizang Plateau, Chengdu Institute of Biology, Chinese Academy of Sciences, Chengdu 610213, China Key Laboratory of Plant Resources Conservation and Sustainable Utilization, South China Botanical Garden, Chinese Academy of Sciences Guangzhou China; 2 University of Chinese Academy of Sciences, Beijing 100049, China Key Laboratory for Plant Diversity and Biogeography of East Asia, Kunming Institute of Botany, Chinese Academy of Sciences Kunming China; 3 Xishuangbanna Tropical Botanical Garden, Chinese Academy of Sciences, Mengla 666303, China Xishuangbanna Tropical Botanical Garden, Chinese Academy of Sciences Mengla China; 4 College of Forestry, Southwest Forestry University, Kunming 650224, China South China National Botanical Garden Guangzhou China; 5 Key Laboratory of Plant Resources Conservation and Sustainable Utilization, South China Botanical Garden, Chinese Academy of Sciences, Guangzhou 510650, China College of Forestry, Southwest Forestry University Kunming China; 6 South China National Botanical Garden, Guangzhou 510650, China China-Croatia Belt and Road Joint Laboratory on Biodiversity and Ecosystem Services, Mountain Ecological Restoration and Biodiversity Conservation Key Laboratory of Sichuan Province, Key Laboratory of National Forestry and Grassland Administration on Biodiversity Conservation on the Qinghai-Xizang Plateau, Chengdu Institute of Biology, Chinese Academy of Sciences Chengdu China; 7 Ningbo Botanical Garden, Ningbo 315201, China University of Chinese Academy of Sciences Beijing China; 8 Key Laboratory for Plant Diversity and Biogeography of East Asia, Kunming Institute of Botany, Chinese Academy of Sciences, Kunming 650201, China Ningbo Botanical Garden Ningbo China

**Keywords:** Anning, legume, Leguminosae, morphology, taxonomy

## Abstract

A new species, *Campylotropis anningensis* L.S. Jiang, E.D. Liu & Bo Xu bis, is described and illustrated. It is similar to *C. argentea*, *C. pinetorum*, and *C. sulcata*, but can be distinguished by its ascending white-pubescent branchlets; ovate, ovate-lanceolate to linear-lanceolate stipules; elliptic, narrowly elliptic to narrowly elliptic-lanceolate leaflets with appressed white-sericeous hairs on the abaxial surface; axillary and terminal racemes with white-pubescent and glandular hairs; lanceolate bracts; longer standard and wings; vexillary stamen connate to the staminal tube only at the base; and obliquely ovate legumes that are densely ascending-pilose. Phylogenomic analysis based on 1,233 single-copy nuclear genes (SCGs) confirms that this new species is distinct from morphologically similar taxa. Conservation status of this species is assessed as Least Concern (LC) according to IUCN Red List criteria.

## Introduction

The genus *Campylotropis*Bunge (1835) is a member of the subtribe Lespedezinae, tribe Desmodieae (Benth.) Hutch., within the subfamily Papilionoideae of Fabaceae (Ohashi et al. 1981; LPWG 2017). Recent taxonomic updates — including the description of two new species (Jiang and Xu 2021; Jiang et al. 2024) and a status revision (Liao and Xu 2020) — have increased the genus to approximately 40 species and 11 infraspecific taxa of shrubs or subshrubs. The genus occurs across temperate and subtropical regions of East Asia, centering in the Hengduan Mountains and southern China. China harbors 35 species, 20 of which are endemic (Fu 1987; Iokawa and Ohashi 2002a, 2002b, 2002c, 2008; Huang et al. 2010; Jiang et al. 2025). *Campylotropis* is characterized by 1-flowered nodes in the racemes, usually caducous bracts; pedicels articulate below the calyx, and a nearly falcate keel with a beak-like acute apex (Fu 1987; Nemoto et al. 1995; Iokawa and Ohashi 2002a, 2002b, 2002c; Huang et al. 2010). It is morphologically similar to *Lespedeza*Michaux (1803) and *Kummerowia*Schindler (1912) in having 1-jointed, 1-seeded, non-glochidiate legumes (Ohashi et al. 1981, 2010; Iokawa and Ohashi 2002a, 2002b, 2002c; Huang et al. 2010). However, it can be distinguished from *Lespedeza* by having a single flower (vs. two flowers) per subtending bract and a falcate keel with a beak-like acute apex (vs. keel not falcate, obtuse at apex) and from *Kummerowia* by its subulate (vs. ovate) stipules and arcuate leaflet lateral veins that do not reach the margin (vs. straight lateral veins extending directly to the margin) (Iokawa and Ohashi 2002a, 2002b, 2002c; Huang et al. 2010; Ohashi et al. 1981) — differences consistent with molecular phylogenetic results (Xu et al. 2012; Jin et al. 2019; Feng et al. 2022; Jiang et al. 2024, 2025). Owing to rapid radiation and morphological plasticity, species of this genus are best identified by a combination of distinctive features (Jiang et al. 2025), including branchlet shape, the presence or absence of stipels and glandular hairs, inflorescence structure, and pod morphology (Iokawa and Ohashi 2002a, 2002b, 2002c; Huang et al. 2010; Ohashi et al. 1981).

During field investigations conducted between 2014 and 2026, we encountered an unidentified *Campylotropis* species from Anning City, Yunnan Province, China, which differs distinctly from all known species. Following a comprehensive literature review (Fu 1987; Iokawa and Ohashi 2002a, 2002b, 2002c; Huang et al. 2010), specimen examination, morphological comparisons, and phylogenomic analysis (Jiang et al. 2025), we conclude that this entity represents a previously undescribed species. It is described and illustrated herein as *Campylotropis anningensis* L.S. Jiang, E.D. Liu & Bo Xu bis, sp. nov.

## Materials and method

Specimens of the new species were collected from Anning City, Yunnan Province, China, and deposited in CDBI (Herbarium of Chengdu Institute of Biology, Chinese Academy of Sciences), HITBC (Herbarium of Xishuangbanna Tropical Botanical Garden, Chinese Academy of Sciences), KUN (Herbarium of Kunming Institute of Botany, Chinese Academy of Sciences), and NPH (Ningbo Botanical Garden Herbarium). Herbarium acronyms follow Thiers (2025, continuously updated). Its habitat, growth form, and fresh vegetative and reproductive structures were photographed and documented. Morphological characters were measured and described based on both living plants and preserved specimens with complete reproductive structures. Terminology follows Beentje (2016). The selection of materials and methods for the phylogenomic analysis followed our previous study (Jiang et al. 2025).

## Results and discussion

*Campylotropis anningensis* most closely resembles taxa with dense tawny or white hairs on abaxial leaflet surfaces, namely *C. argentea*, *C. pinetorum*, and *C. sulcata*, which belong to Clade E in the phylogenetic analysis of Jiang et al. (2025). However, it differs from these species in several morphological characters, including ascending white-pubescent branchlets; ovate, ovate-lanceolate to linear-lanceolate stipules; elliptic, narrowly elliptic to narrowly elliptic-lanceolate leaflets with appressed white-sericeous hairs on the abaxial surface; axillary and terminal racemes with a white-pubescent indumentum intermixed with glandular hairs; lanceolate bracts; and obliquely ovate, densely ascending-pilose legumes.

The phylogenetic trees inferred using Maximum likelihood (ML) and Bayesian inference (BI) based on 1,233 single-copy nuclear genes (SCGs) show identical topologies with high support values (see Fig. 3 in Jiang et al. 2025). These results revealed that the putative new species, *C. anningensis* (as sample *C.* sp._S1119; see Fig. 3 in Jiang et al. 2025), is closely related to other taxa with dense white or tawny indumentum on the abaxial surface of leaflets, all within Clade E. Although *C. anningensis* exhibits the closest morphological resemblance to *C. argentea*, it forms a distinct phylogenetic clade and is not recovered as a sister group to *C. argentea*. Notably, our analyses also strongly support a close relationship among *C. anningensis*, *C. pinetorum* (including subsp. pinetorum and subsp. velutina), and *C. sulcata*, with *C. anningensis* being recovered as sister to the *C. pinetorum* clade. Both morphological and phylogenetic evidence thus support its position in Clade E.

### Taxonomic treatment

#### Campylotropis anningensis

Taxon classificationPlantaeFabalesFabaceae

L.S. Jiang, E.D. Liu & Bo Xu bis sp. nov.

85B278EF-1595-524B-85D6-EB9E14AD375E

urn:lsid:ipni.org:names:77387600-1

Figs 1, 2, 3

##### Diagnosis.

*Campylotropis anningensis* is morphologically most similar to *C. argentea*, but can be distinguished by the following combination of characters: branchlets ascending white-pubescent (vs. appressed white-sericeous), stipules ovate, ovate-lanceolate to linear-lanceolate (vs. lanceolate-linear), leaflets elliptic, narrowly elliptic to narrowly elliptic-lanceolate (vs. elliptic to oblong) with appressed white-sericeous hairs (vs. appressed whitish or silvery silky hairs) on the abaxial surface, inflorescence axillary and terminal racemes (vs. inflorescence axillary racemes and terminal panicles) bearing white-pubescent and glandular hairs (vs. short hairs), bracts lanceolate (vs. ovate-lanceolate), longer standard (10.7–13 mm vs. 9–10 mm) and wings (10–12 mm vs. ca. 10 mm), vexillary stamen connate to the staminal tube only at the base (vs. at the basal 1/3), legumes obliquely ovate (vs. obliquely oblong) that are densely ascending-pilose (vs. sparsely appressed-short hairs).

**Figure 1.**
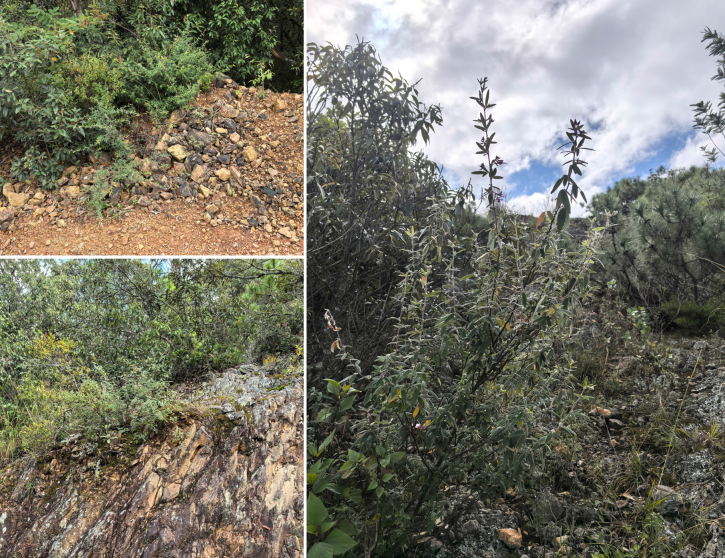
Habitat of *Campylotropis anningensis*. Photographed by Cheng-Wu Liu.

**Figure 2.**
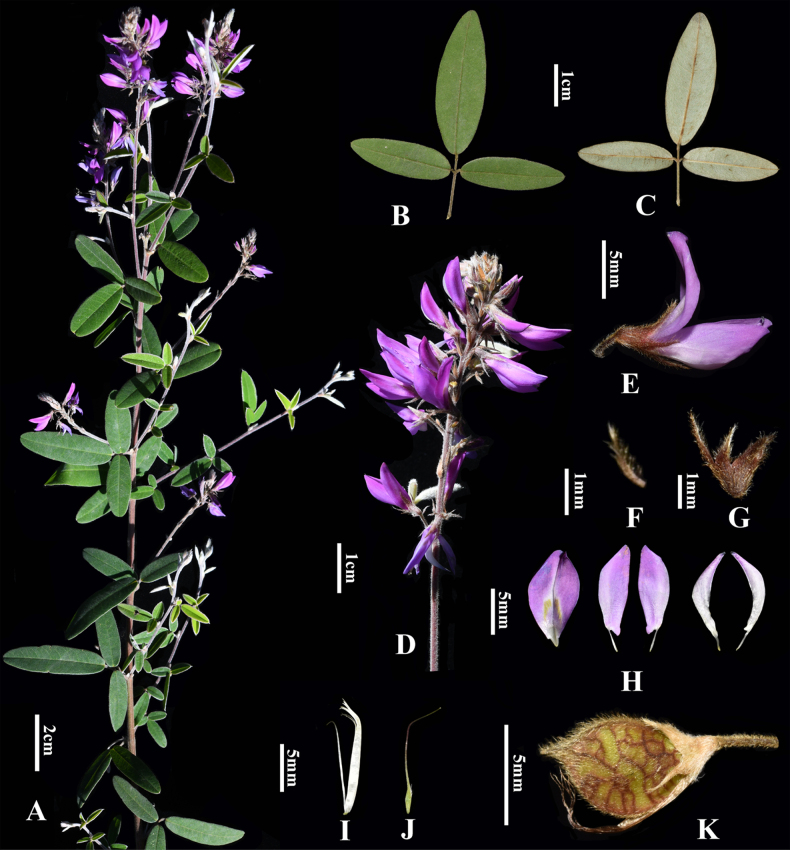
*Campylotropis anningensis*. **A**. Branch and inflorescences; **B**. Leaf (adaxial); **C**. Leaf (abaxial); **D**. Raceme, showing rachis, peduncle, and pedicels; **E**. Flower; **F**. Bracteole; **G**. Calyx; **H**. Standard, wings, and keels; **I**. Stamens; **J**. Pistil; **K**. Legume. **A, D** by En-De Liu; **B, C**, and **E–K** by Li-Sha Jiang.

**Figure 3.**
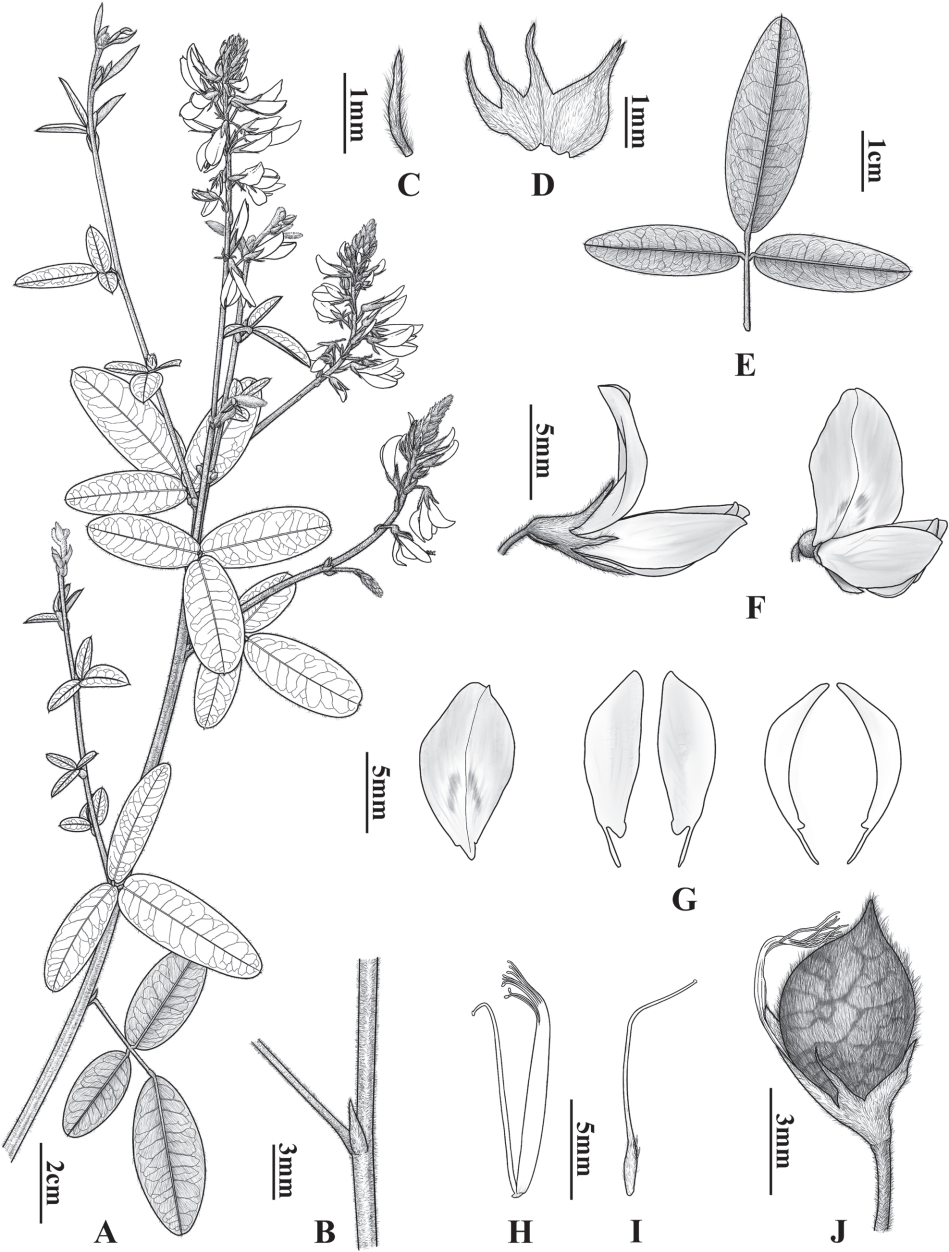
*Campylotropis anningensis*. **A**. Branch and inflorescences; **B**. Branchlet and stipule; **C**. Bracteole; **D**. Calyx; **E**. Leaf (abaxial); **F**. Flower (side and front views); **G**. Standard, wings, and keels; **H**. Stamens; **I**. Pistil; **J**. Legume. Illustration by Yi Wang.

##### Type.

China • Yunnan: Anning City, Xianjie Subdistrict, in grasslands on hillsides, elev. 2,035 m, 28 September 2021, *E. D. Liu, H. J. Dong, J. Zou, C. H. Wang & Y. K. Wang LED11992* (holotype: CDBI!; isotypes: CDBI!, KUN!, NPH!)

##### Description.

Shrubs or subshrubs, erect, 0.8–1.5 m tall. ***Branches*** terete, finely ribbed, obscurely sulcate and densely pubescent with ascending, white hairs when young, glabrescent with age. ***Stipules*** ovate, ovate-lanceolate to linear-lanceolate, 2–5.2 × 0.5–1.7 mm, scarious, persistent. ***Leaves*** pinnately 3-foliolate, 1.4–7.6 cm long, lower ones conspicuously larger than upper ones; petioles 2.5–27 mm, densely ascending white-pubescent; rachis 1–10 mm, indumentum as petioles. ***Leaflets*** slightly coriaceous, elliptic, narrowly elliptic to narrowly elliptic-lanceolate, abaxially densely white-sericeous, sparsely puberulent adaxially, acute or rounded and mucronulate at the apex, obtuse or rounded at the base; midvein and lateral veins prominent abaxially; tertiary veins finely reticulate; terminal leaflet 1.1–5.7 × 0.4–2 cm, lateral ones 0.7–4 × 0.25–1.4 cm; stipels absent. ***Inflorescences*** racemose, axillary and terminal, 2.5–10 cm long, peduncles 0.6–3.5 cm long; flowers sparsely arranged. ***Bracts*** caducous, lanceolate, 2–4 × 0.5–0.9 mm; bracteoles usually caducous but sometimes persistent, linear, 1–1.8 × 0.2–0.4 mm. ***Pedicels*** 2–5 mm long, rachis and pedicels densely ascending white-pubescent mixed with glandular hairs. ***Calyx*** campanulate, deeply 4-lobed, densely ascending white-pubescent mixed with glandular hairs, tube 1.8–2.7 mm; lobes narrowly triangular and acuminate, 2.3–3.4 (–3.7) mm, longer than the tube, lower lobe longest, upper lobes connate except at apex. ***Corolla*** reddish purple; standard elliptic or obovate, 10.7–13 × 5–6 mm, apex obtuse, claw 2–3 mm; wings sub-semi-elliptic, 10–12 × 2.7–3.4 mm, auricle ca. 1 mm long, claw 2–3.3 mm; keel white with purple tip, inflexed at an obtuse angle, 9–12 × 1–2 mm, claw 2–3.5 mm, auricle ca. 0.5 mm long. ***Stamens*** pseudomonadelphous, 9.5–12 mm long, staminal tubes 7.5–9 mm long, vexillary stamen connate to staminal tube only at base (ca. 0.5–0.7 mm). ***Pistil*** 10–13 mm long; ovary 2–3 mm, narrowly ovate, with short hairs; style incurved, 7.3–8.6 mm long. ***Legume*** 1-seeded, obliquely ovate, densely ascending-pilose, with brown reticulate veins, 6–8 × 3–5 mm (immature); apex acute, beaked, 1–1.5 mm long. **Seeds** not seen.

##### Phenology.

Flowering from September to December; fruiting from December to February of the following year.

##### Distribution and habitat.

**Figure 4.**
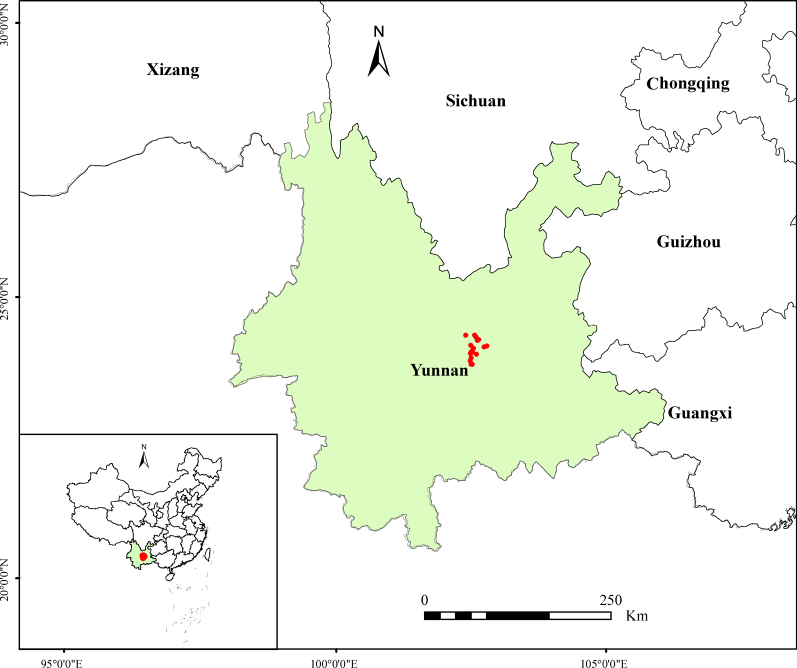
Distribution map of *Campylotropis anningensis* (red circles indicate the voucher locations).

*Campylotropis anningensis* is currently known only from Anning City, Yunnan Province (Fig. 4). It was observed growing in thickets at the margins of valley forests and on grassy slopes at elevations from 1,900 to 2,500 m.

##### Etymology.

The specific epithet *anningensis* refers to the toponym of the type locality, Anning City.

##### Vernacular name.

The Chinese name is proposed here as 安宁杭子梢 (ān níng háng zǐ shāo).

##### Conservation status.

According to the field survey by Dr. En-De Liu et al., this species is frequent along many mountain ridges, forest margins, valleys, and slopes in Anning City, therefore the assessment is categorized as Least Concern (LC) according to the IUCN Red List Categories and Criteria (IUCN 2024). It is likely to be discovered in nearby counties, and further field work is still needed.

##### Additional specimens examined

**(*paratypes*). China** • **Yunnan. Anning City**: Qinglongxia, 15 November 2014, *L. C. Yan 769* (HITBC); • Bajie Subdistrict, Taiyangzhai, Monande Village Committee, elev. 2,330 m, 14 April 2021, *E. D. Liu et al. LED10417* (KUN); • Bajie Subdistrict, Dashiya Village, elev. 2,021 m, 19 April 2021, *E. D. Liu et al. LED10556* (KUN); • Xianjie Subdistrict, Banjia Pass to Heifengshan, elev. 2,494 m, 20 April 2021, *E. D. Liu et al. LED10635* (KUN); • Xianjie Subdistrict, Xiayuanliang Village Committee, Zhongyuanliang, the Chinese Douglas Fir Protected Plot, elev. 1,933 m, 21 April 2021, *E. D. Liu et al. LED10672* (KUN); • Xianjie Subdistrict, Xiayuanliang Village Committee, Zhongyuanliang, the Chinese Douglas Fir Protected Plot, Shangfangshan, elev. 2,001 m, 21 April 2021, *E. D. Liu et al. LED10694* (KUN); • Jinfang Subdistrict, Xiaohebian, elev. 1,909 m, 23 April 2021, *E. D. Liu et al. LED10761* (KUN); • Qinglong Subdistrict, Baguashan, elev. 2,313 m, 10 June 2021, *E. D. Liu et al. LED11402* (KUN); • Lupiao Subdistrict, elev. 2,278 m, 23 September 2021, *E. D. Liu et al. LED11771* (KUN); • Bajie Subdistrict, Monande Reservoir, elev. 1,919 m, 24 September 2021, *E. D. Liu et al. LED11826* (KUN); • Taiping Subdistrict, Daqingshan, elev. 2,259 m, 27 September 2021, *E. D. Liu et al. LED11955* (KUN); • Wenquan Subdistrict, Qiumuyuan, elev. 2,002 m, 13 June 2022, *E. D. Liu et al. LED12932* (KUN); • Laoyangqing to Xiaoqingkou Reservoir, along streams, elev. 2,031 m, 1 November 2023, *E. D. Liu & X. J. Xu LED18325* (NPH); • Xianjie Subdistrict, elev. 2,188 m, 16 October 2024, *X. Li & C. W. Liu JLS0007* (CDBI); • Xianjie Subdistrict, on the way from Heifengshan to Banjia Pass, elev. 2,323 m, 23 October 2025, *E. D. Liu & X. J. Xu LED20888* (KUN); • Xianjie Subdistrict, Heifengshan to Banjia Pass, elev. 2,443 m, 23 October 2025, *E. D. Liu & X. J. Xu LED20952* (KUN); • Xianjie Subdistrict, on the way from Heifengshan to Banjia Pass, elev. 2,449 m, 23 October 2025, *E. D. Liu & X. J. Xu LED20911* (KUN); • Xianjie Subdistrict, Heifengshan to Laoyangqing, elev. 2,148 m, 5 January 2026, *E. D. Liu et al. LED21851* (KUN); • Xianjie Subdistrict, Heifengshan to Laoyangqing, elev. 2,179 m, 5 January 2026, *E. D. Liu et al. LED21846* (KUN); • Bajie Subdistrict, Sancha Reservoir, elev. 2,016 m, 23 March 2026, *X. J. Xu et al. LED21968* (KUN); • Bajie Subdistrict, Laokanshan, elev. 2,298 m, 25 March 2026, *X. J. Xu et al. LED21996* (KUN); • Caopu Subdistrict, Laoyingshan, elev. 2,359 m, 26 March 2026, *X. J. Xu et al. LED22039* (KUN); • Qinglong Subdistrict, Qinglong Village, Leduo, elev. 2,365 m, 31 March 2026, *X. J. Xu et al. LED22094* (KUN); • Wenquan Subdistrict, Longshan to Observation Tower, elev. 1,977 m, 31 March 2026, *X. J. Xu et al. LED22111* (KUN).

##### Morphological comparison.

Morphological comparison between the new species and its relatives reveals that *C. anningensis* most closely resembles *C. argentea*. Nevertheless, it can be readily distinguished from *C. argentea*, as well as from its phylogenetically most closely related taxa (C. pinetorum subsp. pinetorum, C. pinetorum subsp. velutina, and *C. sulcata*), by several characters, including trichome type, inflorescence type, bract shape, and the length of vexillary stamen connation to the staminal tube, among other characters summarized in Table 1.

**Table 1.** Detailed comparison among *Campylotropis anningensis* and its related closely species.

**Character**	***C. anningensis***	***C. argentea***	***C. pinetorum***	***C. sulcata***
**Branchlet shape**	Finely ribbed	Finely ribbed	Angular	Multi-angular, often sulcate
**Branchlet indumentum**	Ascending white-pubescent	Appressed white-sericeous	Ascending tawny-velutinous	Appressed tawny-hairy
**Stipule shape**	Ovate, ovate-lanceolate to linear-lanceolate	Lanceolate-linear	Triangular lanceolate or narrowly triangular	Lanceolate
**Leaflet shape**	Elliptic, narrowly elliptic to narrowly elliptic-lanceolate	Elliptic to oblong	Ovate to elliptic or oblong to narrowly ovate	Elliptic to oblong
**Leaf abaxial indumentum**	Appressed white-sericeous	Appressed whitish or silvery silky hairs	Subappressed tawny- velutinous	Appressed tawny- sericeous
**Inflorescence type**	Axillary and terminal racemes	Axillary racemes and terminal panicles	Axillary and terminal panicles	Axillary and terminal panicles, rarely axillary racemes
**Inflorescence indumentum**	White-pubescent mixed with glandular hairs	Short hairs	Velutinous mixed with dense glandular hairs	Pubescent mixed with glandular hairs
**Bract shape**	Lanceolate	Ovate-lanceolate	Triangular or narrowly triangular	Ovate-lanceolate
**Corolla color**	Reddish purple	Reddish purple	Pinkish white or white	Reddish purple
**Standard length**	10.7–13 mm	9–10 mm	9–10.5 mm	9–10 mm
**Wing length**	10–12 mm	Ca. 10 mm	7.5–9 mm	8.5–9 mm
**Length of vexillary stamen connation to staminal tube**	Only at base	One-third	One-fifth to two-thirds	One-fourth
**Legume shape**	Obliquely ovate	Obliquely oblong	Obliquely elliptic to narrowly obovate	Obliquely elliptic
**Legume indumentum**	Densely ascending-pilose	Sparsely appressed-short hairs	Densely or sparsely ascending-short hairy sometimes mixed with glandular hairs	Sparsely ascending-short hairy

## Supplementary Material

XML Treatment for Campylotropis anningensis

## Additional information

### Artificial Intelligence (AI) use

The authors accept full responsibility for the content of the manuscript, including the disclosure of any use of AI.

No AI tools were used in the preparation of this manuscript.

### Author ORCIDs

L.-S. Jiang https://orcid.org/0000-0001-8520-2573

B. Pan https://orcid.org/0000-0002-0038-9664

J.-Y. Zhu https://orcid.org/0009-0004-0281-9818

X. Li https://orcid.org/0000-0002-1450-8679

K.-W. Jiang https://orcid.org/0000-0002-5917-1846

L.-C. Yan https://orcid.org/0000-0003-1256-6258

C.-W. Liu https://orcid.org/0009-0008-9471-8289

E.-D. Liu https://orcid.org/0000-0003-4424-9820

B. Xu https://orcid.org/0000-0003-3507-9321

### Data availability

All of the data that support the findings of this study are available in the main text.

## References

[B1] Beentje H (2016) The Kew Plant Glossary: An Illustrated Dictionary of Plant Terms, 2^nd^ edn. Kew Publishing, Kew, 184 pp.

[B2] Bunge AA (1835) Plantarum mongholico-chinensium, decas prima. Uchenya zapiski Imperatorskogo Kazanskogo Universiteta, Kazan 4: 154–180.

[B3] Feng Y, Gao XF, Zhang JY, Jiang LS, Li X, Deng HN, Liao M, Xu B (2022) Complete chloroplast genomes provide insights into evolution and phylogeny of *Campylotropis* (Fabaceae). Frontiers in Plant Science 13: 895543. 10.3389/fpls.2022.895543 PMC9158520 35665174

[B4] Fu PY (1987) A study of the genus *Campylotropis* Bunge in China. Bulletin of Botanical Research, Harbin 7: 11–55.

[B5] Huang PH, Ohashi H, Iokawa Y (2010) *Campylotropis*. In: Wu CY, Raven PH, Hong DY (Eds) Flora of China (Vol. 10). Science Press, Beijing & Missouri Botanical Garden Press, St. Louis, 292–302.

[B6] Iokawa Y, Ohashi H (2002a) A taxonomic study of *Campylotropis* (Leguminosae) I. Journal of Japanese Botany 77: 179–222. 10.51033/jjapbot.77_4_9591

[B7] Iokawa Y, Ohashi H (2002b) A taxonomic study of *Campylotropis* (Leguminosae) II. Journal of Japanese Botany 77: 251–283. 10.51033/jjapbot.77_5_9602

[B8] Iokawa Y, Ohashi H (2002c) A taxonomic study of *Campylotropis* (Leguminosae) III. Journal of Japanese Botany 77: 315–350. 10.51033/jjapbot.77_6_9615

[B9] Iokawa Y, Ohashi H (2008) *Campylotropis* (Leguminosae) of China, an enumeration and distribution. Journal of Japanese Botany 83: 36–59. 10.51033/jjapbot.83_1_10023

[B10] IUCN (2024) Guidelines for Using the IUCN Red List Categories and Criteria. Version 16. Prepared by the Standards and Petitions Committee. https://www.iucnredlist.org/documents/RedListGuidelines.pdf

[B11] Jiang LS, Xu B (2021) *Campylotropis luquanensis* (Fabaceae: Papilionoideae), a new species from Southwest China. Phytotaxa 524(2): 114–118. 10.11646/phytotaxa.524.2.6

[B12] Jiang LS, Li XH, Li X, Xu B (2024) *Campylotropis xinfeniae* (Fabaceae, Papilionoideae), a new species from Yunnan, China, based on morphological and molecular evidence. Ecology and Evolution 14(5): e11410. 10.1002/ece3.11410 PMC11103638 38770119

[B13] Jiang LS, Feng Y, Zhang JY, Li X, Liao M, Deng HN, Yu Q, Xu B (2025) Phylogenomic framework, biogeography and character evolution of the genus *Campylotropis* (Fabaceae, Papilionoideae). Molecular Phylogenetics and Evolution 202: 108484. 10.1016/j.ympev.2025.108484 41201954

[B14] Jin DP, Choi IS, Choi BH (2019) Plastid genome evolution in tribe Desmodieae (Fabaceae, Papilionoideae). PLoS ONE 14(6): e0218743. 10.1371/journal.pone.0218743 PMC6590825 31233545

[B15] Liao M, Xu B (2020) *Campylotropis albopubescens* stat. nov. (Leguminosae: Papilionoideae: Desmodieae): the only species in the genus reproduced via rootstocks. Phytotaxa 454(3): 226–230. 10.11646/phytotaxa.454.3.5

[B16] LPWG [The Legume Phylogeny Working Group] (2017) A new subfamily classification of the Leguminosae based on a taxonomically comprehensive phylogeny. Taxon 66(1): 44–77. 10.12705/661.3

[B17] Michaux A (1803) Flora Boreali-Americana, Vol. 2. Levrault, Paris, 340 pp.

[B18] Nemoto T, Ohashi H, Tamate H (1995) Phylogeny of *Lespedeza* and its allied genera (Desmodieae, Lespedezinae). In: Crisp M, Doyle JJ (Eds) Advances in Legume Systematics 7. Royal Botanic Gardens, Kew, 351–358.

[B19] Ohashi H, Polhill RM, Shubert BG (1981) Desmodieae. In: Polhill RM, Raven PH (Eds) Advances in Legume Systematics, Part 1. Royal Botanic Gardens, Kew, 292–300.

[B20] Schindler AK (1912) *Kummerowia* Schindler novum genus Leguminosarum. Repertorium specierum novarum regni vegetabilis 10: 403. 10.1002/fedr.19120102410

[B21] Thiers B (2025 [continuously updated]) Index Herbariorum: a global directory of public herbaria and associated staff. New York Botanical Garden’s Virtual Herbarium. http://sweetgum.nybg.org/science/ih

[B22] Xu B, Wu N, Gao XF, Zhang LB (2012) Analysis of DNA sequences of six chloroplast and nuclear genes suggests incongruence, introgression, and incomplete lineage sorting in the evolution of *Lespedeza* (Fabaceae). Molecular Phylogenetics and Evolution 62(1): 346–358. 10.1016/j.ympev.2011.10.007 22032991

